# Microbial composition of carapace, feces, and water column in captive juvenile green sea turtles with carapacial ulcers

**DOI:** 10.3389/fvets.2022.1039519

**Published:** 2022-12-15

**Authors:** Yide Guo, Hualing Chen, Ping Liu, Fumin Wang, Linmiao Li, Mingbin Ye, Wenge Zhao, Jinping Chen

**Affiliations:** ^1^College of Life Science and Technology, Harbin Normal University, Harbin, Heilongjiang, China; ^2^Guangdong Key Laboratory of Animal Conservation and Resource Utilization, Guangdong Public Laboratory of Wild Animal Conservation and Utilization, Institute of Zoology, Guangdong Academy of Sciences, Guangzhou, Guangdong Province, China; ^3^Huidong Sea Turtle National Reserve Management Bureau, Sea Turtle Bay, Huidong, Guangdong, China

**Keywords:** *Chelonia mydas*, 16S rRNA, ITS, ulceration, high-throughput sequencing, *Psychrobacter*

## Abstract

**Introduction:**

Green sea turtles are endangered marine reptiles. Carapacial ulcers will develop on juvenile green sea turtles during artificial rescue, seriously affecting their health and potentially leading to death.

**Methods:**

To determine the pathogens causing ulcerative carapacial disease, we performed 16S and ITS high-throughput sequencing, and microbial diversity analysis on samples from carapacial ulcers, healthy carapaces, feces, and seawater of juvenile green sea turtles.

**Results:**

Our analysis showed that changes in microbial diversity of green sea turtle feces and seawater were not significantly associated with ulcerative carapacial disease.

**Discussion:**

*Psychrobacter* sp. is the dominant species in the carapacial ulcers of green sea turtles. The bacterium is present in both healthy turtles and seawater where carapacial ulcers did not occur and decreasing seawater temperatures are likely responsible for the infection of juvenile green turtles with *Psychrobacter* sp. This is the first study on carapacial ulcers in captive juvenile green sea turtles. Our research provides theoretical guidance for the prevention and control of carapacial ulcers in captive juvenile green sea turtles.

## Introduction

The green sea turtle (*Chelonia mydas*) is one of the rarest marine reptiles worldwide and has been listed in the International Union for Conservation of Nature (IUCN) Red List of Threatened Species as endangered (EN), and the Washington Convention on International Trade in Endangered Species (CITES) as an Appendix I protected animal (1). The Huidong Sea Turtle National Nature Reserve is the largest artificial rescue base for sea turtles in China. Juvenile green turtles are kept in captivity until they are strong enough to be released back into the ocean. In conservation efforts, skin and shell diseases are important factors affecting the health of sea turtles. For example, skin tumors in green sea turtles can significantly affect their ability to forage for food and avoid predation(2, 3). To date, most studies on sea turtle-related diseases have focused on wild rescued individuals and studies on captive sea turtle pathologies are lacking.

Artificially breeding endangered wildlife and releasing them into the natural environment is an effective means of increasing populations. Green turtles were bred and laid in aquariums, and all hatchlings were raised in captivity before being released into the wild. During artificial breeding, diseases can seriously affect the health of individual animals and even lead to death. Recent studies have shown that a captive green sea turtle is susceptible to various diseases (4–6). Viral, fungal, and bacterial infections can cause skin diseases such as ulcerative dermatitis (UD) (4) and conjunctivitis in captive sea turtles (7).

To date, there have been many microbiological studies on sea turtles. Several studies have demonstrated that Chelonid Alphaherpes virus 5 (ChHV5) causes fibropapillomatosis (FP) in the head and extremities of sea turtles, which can lead to restricted movement and reduced feeding ability (8–10). Previous studies have shown that environmental pollution, while correlated with habitat destruction, has no impact on ChHV5. However, environmental pollution will increase the morbidity of FP (11). Furthermore, it has been reported that sea turtles can be infected with various bacteria, including *Aeromonas hydrophila* (12), *Mycobacterium haemophilum* (13), *Mycobacterium chelonae* (14–16), *Staphylococcus* spp. (17), and *Acinetobacter* spp. (18). Such bacterial infections will cause lethal damage to sea turtles.

Our study was to follow up on clinical observations made during the course of practice. The emergence of ulcerative carapacial diseases seriously affects conservation efforts of juvenile green sea turtles. In our previous investigations, we found that captive juvenile green sea turtles often develop carapacial ulcers in autumn and winter. Without treatment, the wound area of the carapacial ulcer gradually expands, and individuals with severe infections die. The disease is also contagious and most individuals in the same pond develop similar symptoms, often within a week. Fortunately, administering amikacin or ceftriaxone sodium intramuscularly can effectively treat the disease. Therefore, the pathogenic bacteria causing carapacial ulcers warrant further investigation. To identify the causative agent of ulcerative carapacial disease in juvenile green sea turtles, we performed microbial diversity analysis on samples of carapacial ulcers, healthy carapaces, feces, and water. The objective of this study was to: (1) characterize microbial diversity in captive juvenile green sea turtles and their environment, and (2) identify potential pathogens causing carapacial ulcers in juvenile green sea turtles.

## Materials and methods

### Study site and sample collection

**Figure 1:**
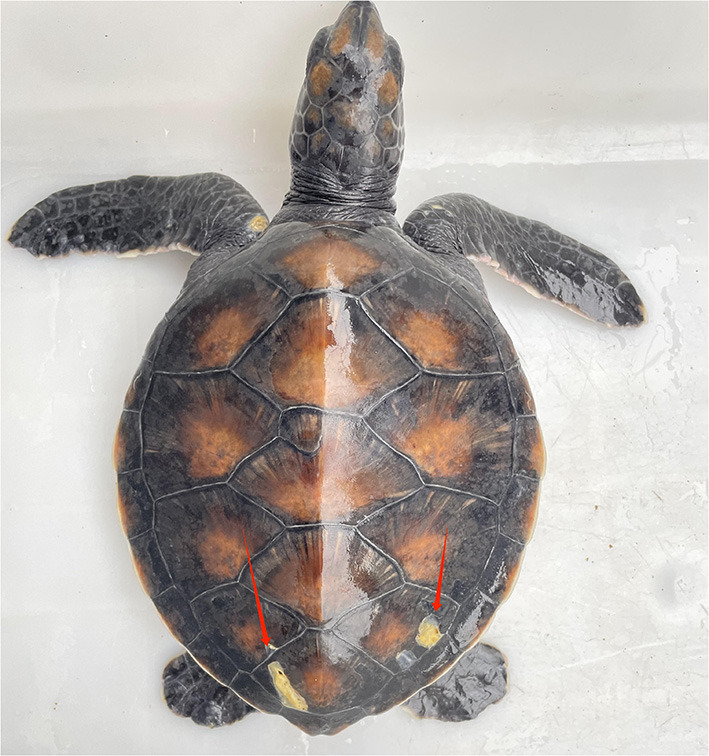
Carapacial ulcer of juvenile green sea turtle.

This study was conducted at the Huidong Sea Turtle National Nature Reserve, Guangdong Province, China (22°33′15″-22°33′20″ E, 114°52′50″-114°54′33″ N), between March and April 2022. All captive green turtles were housed in 13 outdoor concrete pools. Pumps were used to draw seawater directly from the shore, and the seawater was completely replaced every 7 days. All green turtles shared the same diet, which includes turtle compound feed (Zhongshan President company, China), vegetables (lettuce or cabbage), and squid. The staff used heating equipment during the winter months to maintain water temperatures between 25 and 28°C in 10 of the pools, which were continuously monitored by an electronic thermometer. However, due to limitations in the heating equipment, three of the pools were not heated in winter. The temperature of pools without heating in winter was between 12 and 15°C. In this study, captive juvenile green turtles (both sick and healthy) from the Huidong Sea Turtle National Nature Reserve underwent high-throughput sequencing of bacteria and fungi to analyze the main microorganisms causing carapacial ulcers in green turtles (Figure 1).

To obtain microbial diversity in captive sea turtles and their environment, we collected carapacial, fecal, and environmental water samples from juvenile green sea turtles. In addition, because carapacial ulcers have a severe effect on turtle survival, we sampled healthy and diseased individuals separately. Healthy turtles were from heated pools and diseased turtles were from non-heated pools. A total of 38 juvenile sea turtle samples and eight environmental samples were collected, including five healthy shell samples, 18 carapacial ulcer samples, 15 fecal samples (10 from diseased turtles and five from healthy turtles), and eight water samples (five from pools containing turtles with carapacial ulcers and three from pools containing turtles without carapacial ulcers). For comparison, we divided the samples into six groups. Group A, C, and E were from heated pools. Group B, D, and F were from non-heated pool. The groups included carapacial samples from healthy green sea turtles (Group A), carapacial samples from diseased green sea turtles (Group B), fecal samples from healthy green sea turtles (Group C), fecal samples from diseased green sea turtles (Group D), water column samples from pools containing turtles with ulcerative carapacial disease (Group E), and water column samples from pools containing turtles without ulcerative carapacial disease (Group F). The shell surfaces of healthy individuals were rinsed with sterile saline, followed by repeated wiping of the shells of juvenile turtles with sterile flocked swabs 30 times, which were kept in sterile lyophilized tubes. The area around the shell lesion of diseased hatchlings was disinfected using 75% alcohol, after which the ulcer tissue was carefully excised using sterile surgical scissors and preserved in sterile preservation tubes. A sterile swab was used to gently enter the cloaca of the hatchling turtle. The swab was rotated several times to adhere to the fecal sample, which was collected and stored in a sterile preservation tube. The water samples were collected in sterile water collection bags and filtered through 220 nm filter membranes, which were stored in sterile preservation tubes. All samples were quickly stored on dry ice after collection and were brought back to the laboratory for processing. The details of all the samples are presented in Supplementary Table S1.

### DNA extraction, PCR, and Hi seq sequencing

DNA was extracted using the TGuide S96 Magnetic Soil/Stool DNA Kit [Tiangen Biotech (Beijing) Co., Ltd.] according to the manufacturer's instructions. The DNA concentration of the samples was measured using the Qubit dsDNA HS Assay Kit and a Qubit 4.0 Fluorometer (Invitrogen, Thermo Fisher Scientific, Oregon, USA). The 27F: AGRGTTTGATYNTGGCTCAG and 1492R: TASGGHTACCTTGTTASGACTT universal primer sets were used to amplify the full-length 16S rDNA gene from genomic DNA extracted from each sample. The ITS1:5′-CTTGGTCATTTAGAGGAAGTAA-3′ and ITS4:5′-TCCTCCGCTTATTGATATGC-3′ ACTT universal primer sets were used to amplify the full-length ITS gene from the genomic DNA extracted from each sample. Both the forward and reverse 16S and ITS primers were tailed with sample-specific PacBio barcode sequences to allow multiplexed sequencing. We chose to use barcoded primers because this reduces chimera formation compared with the alternative protocol in which primers are added in a second PCR reaction. The KOD One PCR Master Mix (TOYOBO Life Science) was used to perform PCR amplification, with initial denaturation at 95°C for 2 min; followed by 25 cycles of denaturation at 98°C for 10 s, annealing at 55°C for 30 s, and extension at 72°C for 1 min 30 s; and a final step at 72°C for 2 min. The PCR amplicons were purified using Agencourt AMPure XP Beads (Beckman Coulter, Indianapolis, IN, USA) and quantified using the Qubit dsDNA HS Assay Kit and Qubit 4.0 Fluorometer (Invitrogen, Thermo Fisher Scientific, Oregon, USA). After the individual quantification step, the amplicons were pooled in equal amounts. SMRTbell libraries were prepared from amplified DNA using the SMRTbell Express Template Prep Kit 2.0, according to the manufacturer's instructions (Pacific Biosciences). Purified SMRTbell libraries from the pooled and barcoded samples were sequenced on a single PacBio Sequel II 8M cell using Sequel II Sequencing kit 2.0.

### Bioinformatic analysis

The bioinformatics analysis of this study was performed using the BMK Cloud (Biomarker Technologies Co., Ltd., Beijing, China). The raw reads generated from sequencing were filtered and demultiplexed using the SMRT Link software (version 8.0) with min Passes ≥5 and min Predicted Accuracy ≥0.9, to obtain circular consensus sequencing (CCS) reads. Subsequently, lima (version 1.7.0) was employed to assign CCS sequences to the corresponding samples based on their barcodes. CCS reads containing no primers and those reads beyond the length range (1,200–1,650 bp) were discarded through the recognition of forward and reverse primers and quality filtering using the cut adopted quality control process (version 2.7). The UCHIME algorithm (v8.1) was used to detect and remove chimeric sequences to obtain clean reads. Sequences with similarity ≥97% were clustered into the same operational taxonomic unit (OTU) using USEARCH (v10.0), and the OTUs with relative abundance < 0.005% were filtered. Taxonomic annotation of the OTUs was performed based on the naive Bayes classifier in QIIME2 using the SILVA database (release 132) with a confidence threshold of 70%. Alpha diversity was calculated using QIIME2 and displayed using R software. Beta diversity was determined using QIIME to evaluate the degree of similarity of microbial communities from different samples. Principal coordinate analysis (PCoA), heat maps, UPGMA, and non-metric multidimensional scaling (NMDS) were used to analyze beta diversity. Furthermore, we employed linear discriminant analysis (LDA) effect size (LEfSe) to test for significant taxonomic differences among the groups. A logarithmic LDA score of 4.0 was set as the threshold for discriminative features.

## Results

### Overview of the sequencing data

Forty-six samples were sequenced from the bacterial and fungal communities. For the bacterial community, 5,96,230 sequences were obtained, and each sample contained at least 9,959 effective sequences for OTU analysis. This indicated that the sequencing depths were sufficient to capture most bacterial OTUs (Supplementary Figure S1A). In total, 1,275 bacterial OTUs were obtained from all samples, and these OTUs were annotated into 22 bacterial phyla.

For the fungal community analysis, 6,16,231 sequences were obtained, and each sample contained at least 9,445 effective sequences for OTU analysis. The rarefaction curve showed that the sequencing depths were sufficient to capture most fungal OTUs (Supplementary Figure S1B). In total, 1,436 fungal OTUs were obtained from all samples, and these OTUs were annotated into 15 fungal phyla.

### Microbial community structure in green turtle samples and water

**Figure 2:**
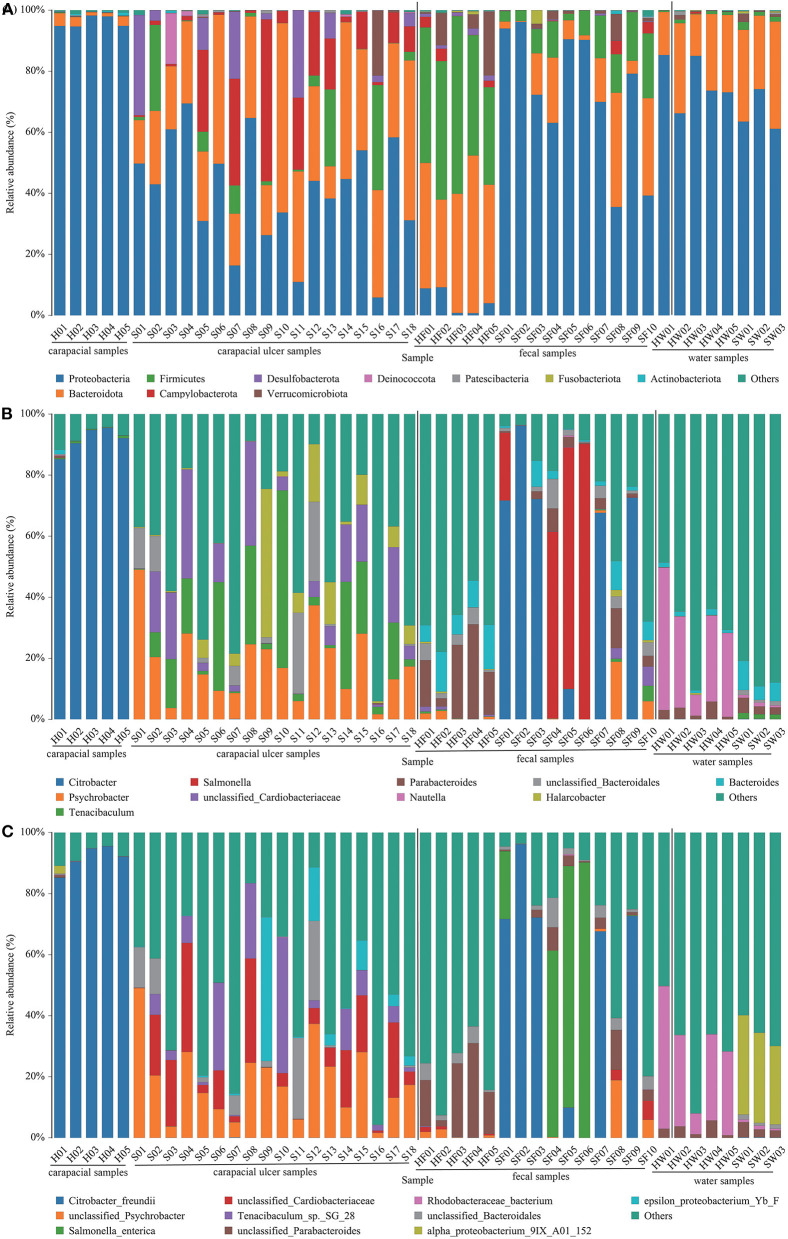
Composition of bacteria in each sample at the phylum **(A)**, genus **(B)**, and species **(C)** level. The taxa with low abundance were included in “others.” Carapacial samples (including ulcer samples), fecal samples and water samples were separated by the black vertical line.

The mean relative abundance of bacteria in all samples were determined. At the phylum level, the predominant bacterial communities in each group were as follows: (1) Proteobacteria (95.73%) was the predominant taxa in group A. (2) Proteobacteria (40.66%) and Bacteroidetes (31.56%) were the predominant taxa in group B. (3) Firmicutes (43.60%) and Bacteroidota (39.73%) were the predominant taxa in group C. (4) Proteobacteria (73.16%), Bacteroidota (13.17%), and Firmicutes (9.92%) were the predominant taxa in group D. (5) Proteobacteria (76.64%) and Bacteroidota (21.56%) were the predominant taxa in group E. (6) Proteobacteria (66.06%) and Bacteroidetes (29.95%) were the predominant taxa in group F (Figure 2A, Supplementary Table S2). The main bacteria in each group at the genus level are shown in Figure 2B, Supplementary Table S2. At the species level, the bacterial communities were as follows: (1) *Citrobacter freundii* (91.44%) was the predominant taxon in group A. (2) Unclassified *Psychrobacter* sp. (18.34%), unclassified *Cardiobacteriaceae* sp. (11.82%), and *Tenacibaculum* sp. SG-28 (8.42%) were the predominant taxa in group B. (3) Unclassified *Parabacteroides* sp. (17.22%) and unclassified *Lachnospiraceae* sp. (7.26%) were the predominant taxa in group C. (4) *Citrobacter freundii* (39.16%) and *Salmonella enterica* (25.31%) were the predominant taxa in group D. (5) *Rhodobacteraceae* bacterium (27.73%), *Phaeobacter gallaeciensis* (16.94%), and *Rhodobacterales* bacterium CB1079 (15.78%) were the predominant taxa in group E. (6) alpha proteobacterium 9IX/A01/152 (29.51%) and marine alpha proteobacterium AS-21 (14.24%) were the predominant taxa in group F (Figure 2C, Supplementary Table S2).

**Figure 3:**
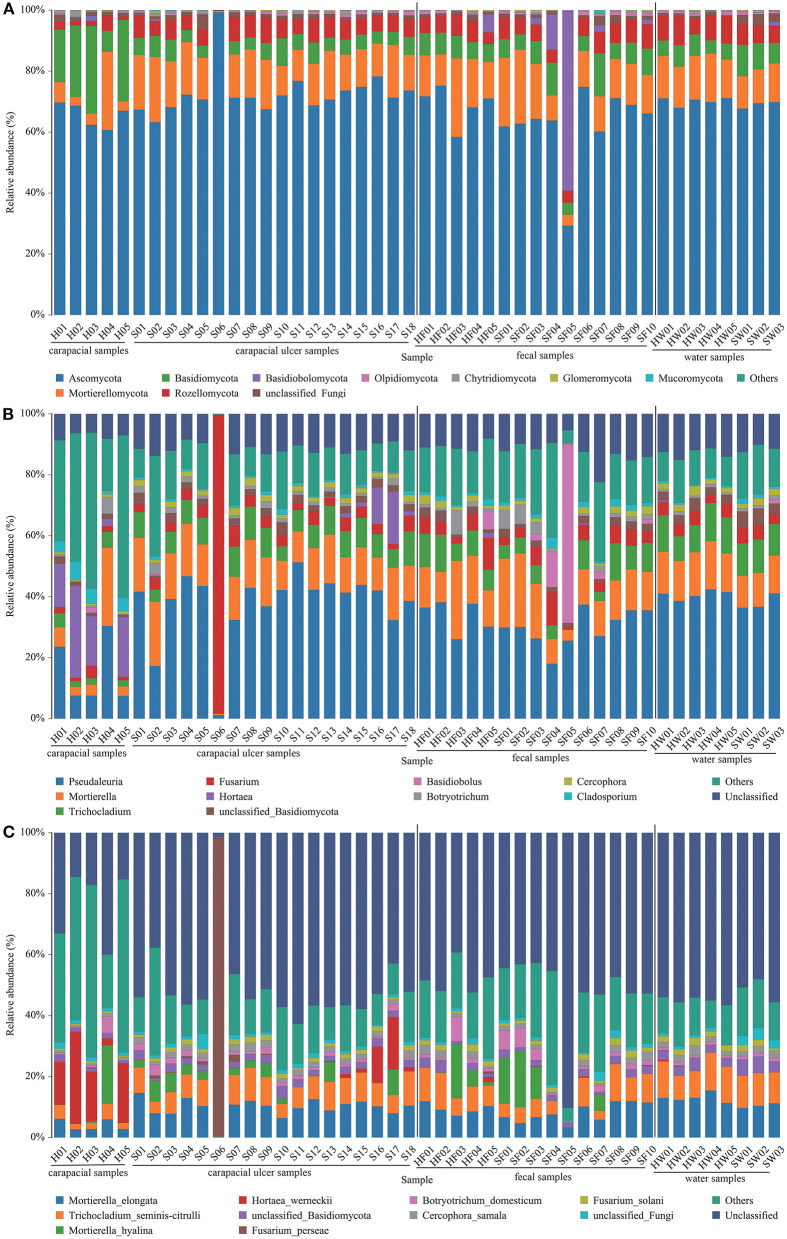
Composition of fungus in each sample at the phylum **(A)**, genus **(B)**, and species **(C)** level. The taxa with low abundance were included in “others.” Carapacial samples (including ulcer samples), fecal samples, and water samples were separated by the black vertical line.

The mean relative abundance of fungi in all samples were determined. At the phylum level, the fungus communities were: (1) Ascomycota (65.67%) and Basidiomycota (20.55%) were the predominant taxa in group A. (2) Ascomycota (72.82%) and *Mortierellomycota* (13.69%) were the predominant taxa in group B. (3) Ascomycota (68.89%) and *Mortierellomycota* (15.52%) were the predominant taxa in group C. (4) Ascomycota (62.31%) and *Mortierellomycota* (13.94%) were the predominant taxa in group D. (5) Ascomycota (70.13%) and *Mortierellomycota* (14.08%) were the predominant taxa in group E. (6) Ascomycota (68.99%) and *Mortierellomycota* (11.55%) were the predominant taxa in group F (Figure 3A, Supplementary Table S2). The main fungi in each group at the genus level are shown in Figure 3B, Supplementary Table S2. At the species level, the fungus communities were: (1) Unclassified (24.01%) and *Hortaea werneckii* (16.53%) were the predominant taxa in group A. (2) Unclassified (50.32%), *Mortierella elongate* (9.77%), and *Trichocladium yaline*-*citrulli* (7.66%) were the predominant taxa in group B. (3) Unclassified (47.89%), *Mortierella elongata* (9.42%), and *Trichocladium yaline*-*citrulli* (8.74%) were the predominant taxa in group C. (4) Unclassified (52.42%) and *Mortierella hyaline* (8.03%) were the predominant taxa in group D. (5) Unclassified (55.06%), *Mortierella elongata* (13.03%), and *Trichocladium seminis*-*citrulli* (10.65%) were the predominant taxa in group E. (6) Unclassified (51.45%), *Mortierella elongata* (10.43%), and *Trichocladium seminis*-*citrulli* (10.55%) were the predominant taxa in group F (Figure 3C, Supplementary Table S2).

### Microbial diversity analysis of sea turtles and environmental samples

**Figure 4:**
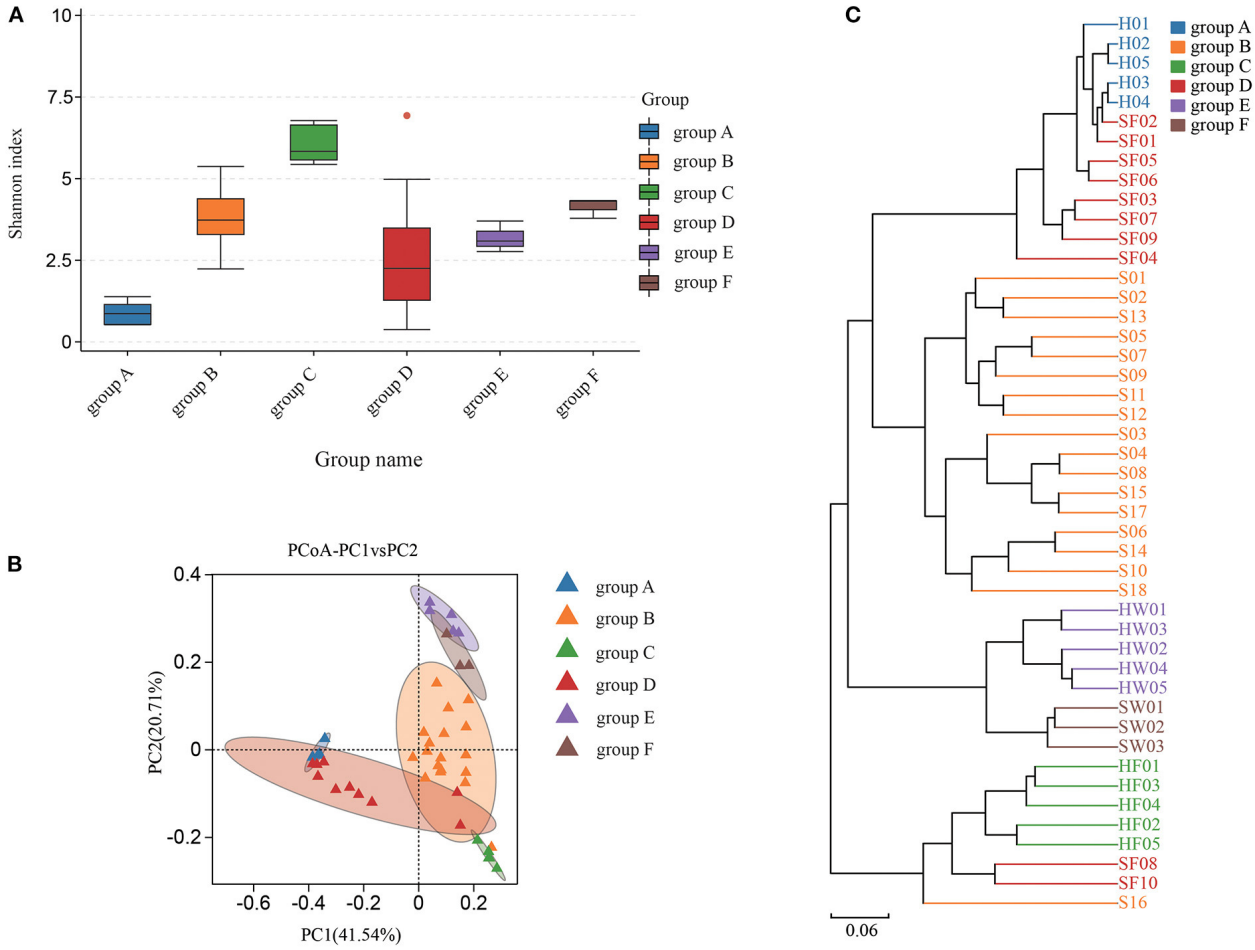
Community diversity of bacterial microbiome for each group. **(A)** Alpha diversity is indicated by the Shannon index. **(B)** Beta diversity is indicated by principal coordinate analysis (PCoA) and based on the weighted UniFrac distance matrix. **(C)** Beta diversity is indicated by the UPGMA cluster and based on the weighted UniFrac distance matrix. Group A: Carapacial samples from healthy green turtles. Group B: Carapacial ulcer samples from diseased green turtles. Group C: Fecal samples from healthy green turtles. Group D: Fecal samples from diseased green turtles. Group E: Water samples from pools without carapacial ulcers. Group F: Water samples from pools with carapacial ulcers.

**Figure 5:**
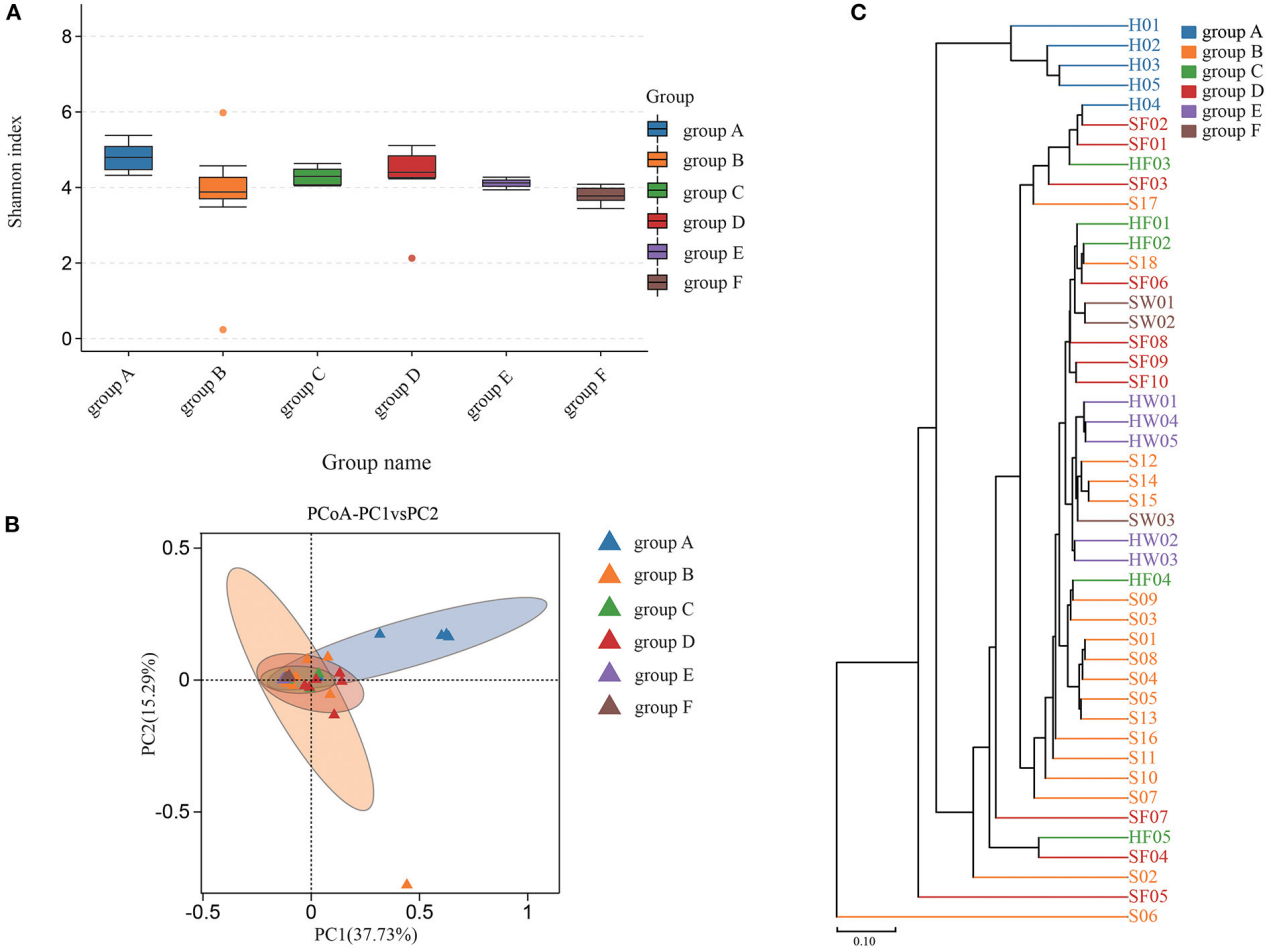
Community diversity of fungus microbiome for each group. **(A)** Alpha diversity is indicated by the Shannon index. **(B)** Beta diversity is indicated by principal coordinate analysis (PCoA) and based on the Bray-Curtis distance matrix. **(C)** Beta diversity is indicated by the UPGMA cluster and based on the Bray-Curtis distance matrix. Group A: carapacial samples from healthy green turtles. Group B: carapacial ulcer samples from diseased green turtles. Group C: fecal samples from healthy green turtles. Group D: fecal samples from diseased green turtles. Group E: water samples from pools without carapacial ulcers. Group F: water samples from pools with carapacial ulcers.

Shannon index was used to estimate the alpha diversity. The Shannon index, ranging from large to small, were group C, group F, group B, group E, group D, and group A. A higher Shannon index value indicated higher community diversity (Figure 4A). For beta diversity, PCoA (Figure 4B) and UPGMA clustering (Figure 4C) showed that the six groups clustered differently. The PERMANOVA test also supported this clustering (*P* < 0.05) (Supplementary Table S3). The Shannon index of group B was higher than that of group A, which indicated that there was in increase in some species of bacteria in group A. Indeed, eight bacterial species were significantly increased in group A. Compared with groups C and D, eight bacterial species were significantly increased in group C and one bacterial species was increased in group D. The water samples revealed that nine bacterial species were significantly increased in group F, while six bacteria were increased in group E (Supplementary Figure S4). Based on the KEGG database, the functions of the 46 samples included metabolism, environmental information processing, genetic information processing, cellular processes, human diseases, and organismal systems at Level 1 (Supplementary Figure S2). Furthermore, the changes in function between samples from healthy animals and samples from diseased animals were confirmed at level 2 (Supplementary Figure S3). Regarding the community diversity of fungi, the comparison of the Shannon index showed that the alpha diversity in group A was significantly higher than that in group B. However, there was no significant difference in alpha diversity in fecal and water samples (Figure 5A). For beta diversity, the results of PCoA (Figure 5B), UPGMA clustering (Figure 5C), and PERMANOVA were consistent, showing significant differences in the diversity of the fungal communities between samples from healthy and diseased green sea turtles (*P* < 0.05) (Supplementary Table S3). However, there was no significant difference in beta diversity between groups C and D (*P* value greater than 0.05) (Supplementary Table S3). In addition, two fungi were significantly increased in group B and seven fungi were increased in group A. As for the water samples, one fungus significantly increased in groups E and F (Supplementary Figure S5).

### Opportunistic pathogens that may cause green turtle carapacial ulcers

Unclassified *Psychrobacter* sp. and unclassified *Coriobacteriaceae* sp. were present in all carapacial ulcer samples, with mean relative abundances of 18.34 and 11.82%, respectively (Figure 2B). Interestingly, unclassified *Psychrobacter* sp. and unclassified *Coriobacteriaceae* sp. were present in samples other than the carapacial ulcer samples. Unclassified *Psychrobacter* sp. were detected in 40% of healthy carapace samples, 80% of fecal samples from healthy green turtles, 70% of fecal samples from diseased green turtles, 33% of water samples from pools with ulcerative carapacial disease, and 40% of water samples from pools without ulcerative carapacial disease (Supplemental Table S2). Unclassified *Coriobacteriaceae* sp. were detected in 80% of healthy carapace samples, all fecal samples from healthy green turtles, 70% of fecal samples from diseased green turtle, 33% of water samples from pools with ulcerative carapacial disease, and 40% of water samples from pools without ulcerative carapacial disease (Supplementary Table S2).

## Discussion

Based on personal observations, we noticed that the ulcerative carapacial disease occurs mostly in autumn and winter. In winter, the water temperature will drop to between 15 and 20°C, while in summer, it averages at ~28°C. Our field research suggests that not all captive green sea turtles develop ulcerative carapacial disease, which may be related to the pond environment. During the onset of the disease, the water temperature in the farming pond where the disease occurs is low, and the turtles display decreased vitality and feeding. In addition, the decrease in water temperature indirectly causes the turtle to lose weight, but it is not known whether the decrease in weight is related to the occurrence of ulcerative carapacial disease. Because of air heaters, water temperatures in some ponds can be maintained at ~25°C during the winter, and sea turtles in these ponds do not experience decreased vitality, feeding, or weight loss. Green sea turtles older than 1 year rarely experience similar symptoms at similarly low water temperatures, with most cases concentrated on the birth of newborn turtles, which may be associated with increased sea turtle resistance. Considering these observations, we believe that artificial control of water temperature in turtle farming ponds is necessary, and that turtle ulcerative carapacial disease may be relieved as the water is maintained at a higher temperature.

Furthermore, our findings provide information on captive juvenile green sea turtles, including turtle shells, feces, and water column. Most studies on turtle disease have focused on skin tumors (19–22), parasites (23–26), and bacterial infections (27–29), and there are few reports of infectious carapacial ulcers. Our study showed that the main bacterium in juvenile green sea turtle shells was *Citrobacter freundii* (91.44% on average). *Citrobacter freundii* is an important human-animal bacterium that causes food poisoning, diarrhea, and urinary tract infections (30). *Citrobacter freundii* has been found in a variety of animals, including sheep (*Ovis aries*) (31), Chinese sturgeon (*Acipenser sinensis*) (32), silver catfish (*Rhamdia quelen*) (33), loggerhead turtles (*Caretta caretta*) (34–36), and green sea turtles (*Chelonia mydas*) (29). Therefore, this finding was unsurprising.

Turtle carapacial ulcers may have been caused by bacterial infection (37). Our findings suggest that carapacial ulcers have multiple pathogenic bacteria, with the *Psychrobacter* sp. and unclassified *Cardiobacteriaceae* sp. present in all carapacial ulcer samples, which demonstrates the presence of a mixed infection with bacteria in the ulcerated tissue. *Psychrobacter* sp. is an opportunistic pathogen found in a wide range of environments and poses a potential risk of human infection (38). Studies have reported the occurrence of *Psychrobacter* spp. infections in humans after surgery, blood transfusion, or exposure to the marine environment (38–40). A study on Antarctic krill (*Euphausia superba*) showed that secondary infection by *Psychrobacter* sp. occurred after parasitic infection in Antarctic krill (41). In our study, *Psychrobacter* sp. and *Cardiobacteriaceae* sp. were detected in both healthy turtles and the seawater they were housed in, suggesting that the causative agent of carapacial ulcers was environmental. *Psychrobacter* sp. is a psychrotolerant bacterium and low temperatures are conducive to its growth, inferring that it may be an opportunistic pathogen that infects green sea turtles in favorable conditions. In the present study, the highest relative abundance of *Psychrobacter* sp. was found in ulcerated tissues, therefore we speculate that *Psychrobacter* sp. is an opportunistic pathogen causing carapacial ulcers in sea turtles, with multiple bacterial infections occurring after lesion formation. *Cardiobacteriaceae* sp. is an environmental bacterium that is carried in the Gentoo penguin (*Pygoscelis papua*) (42) and striped dolphin (*Stenella coeruleoalba*) (43), but there are no reports of marine organisms having disease caused by this bacterium. Based on existing research, we cannot determine whether *Cardiobacterium* sp. causes carapacial ulcers in juvenile green sea turtles in captivity.

Our findings suggest that there are changes in the gut microbiota of diseased green sea turtles. The mean relative abundance of *Salmonella enterica* in the gut microbes of diseased turtles was significantly higher than that in healthy green turtles; however, this was not significant in a single sample (Figure 2C). Reptiles are reservoirs of *Salmonella* spp. (44, 45), and the discovery of *Salmonella* spp. in green turtles is expected. *Salmonella* is a human-animal pathogen and contact between humans and turtles may lead to *Salmonella* infections in humans (46). Compared with the public, staff involved in sea turtle rescue management are exposed to more serious health risks (47), and practitioners should take necessary measures to prevent potential public health problems. In the aqueous environment samples, the subgroups in which diseased turtles appeared had a significantly higher mean relative abundance of nine bacterial species; however, these nine species did not include the dominant bacteria in the carapacial ulcer samples, nor did they include *Salmonella enterica*. The above analysis suggests that changes in water column bacteria may not be the cause of turtle carapacial ulcers.

A study on loggerhead turtles (*Caretta caretta*) showed that *Fusarium* spp. causes carapacial ulcers in sea turtles, and fungal diagnosis should be added to the diagnosis of ulcerative carapacial disease in sea turtles (48). Our data analysis showed that the average relative abundances of *Mortierella elongata* (9.77%) and *Trichocladium seminis*-*citrulli* (7.66%) were significantly higher in ulcerated tissues than in healthy turtle carapaces. However, these two fungi were not dominant in the ulcer tissue and may not be the causative pathogens. We also analyzed fungal changes in the gut group, and statistical analysis showed no significant changes in the gut fungi of healthy and diseased green turtles. For the water samples, the average relative abundance of unclassified fungi (3.20%) was significantly higher in water samples where the disease occurred than in water samples where the disease did not occur. The average relative abundance of unclassified fungi in the turtle carapace, ulcerated tissue, and gut microbes remained stable, suggesting that changes in environmental water fungi have limited effects on green sea turtles. The results of this study are important for biomonitoring shell ulcer-causing microorganisms in captive juvenile green sea turtles, assessing the threat of pathogenic bacteria in green turtles, and establishing appropriate treatment plans. This is the first time that skin microorganisms and pathogenic microorganisms associated with juvenile green sea turtles in the Huidong Sea Turtle National Nature Reserve have been identified at a molecular level.

Although progress has been made in the study of carapacial ulcers in captive juvenile green sea turtles, our study has some limitations. We did not test whether low temperatures were a factor associated with ulcerative carapacial disease in green turtles. In future studies, we will investigate the role of temperature in the occurrence of ulcerative carapacial disease.

## Conclusion

It should be noted that carapacial ulcers are prevalent in green turtles at lower seawater temperatures, and that measures should be taken to reduce the occurrence of carapacial ulcers in juvenile green sea turtles during the autumn and winter. The opportunistic pathogens that cause carapacial ulcers may be *Psychrobacter* sp. and *Coriobacteriaceae* sp., both of which are present in healthy turtles and seawater samples. Furthermore, the occurrence of carapacial ulcers in turtles may be related to a decrease in the seawater temperature. Basic research on captive green sea turtles and their aquatic environments provides a reference for the protection of this endangered marine creature. Future research should delve into the pathogenic mechanism causing green sea turtle carapacial ulcers in order to gain a deeper understanding of the disease drivers.

## Data availability statement

The datasets presented in this study can be found in online repositories. The names of the repository/repositories and accession number(s) can be found below: https://www.ncbi.nlm.nih.gov/bioproject/PRJNA876638, PRJNA876638 and https://www.ncbi.nlm.nih.gov/bioproject/PRJNA876654, PRJNA876654.

## Ethics statement

The animal study was reviewed and approved by the Committee on the Ethics of Animal Experiments of the Institute of Zoology of Guangdong Academy of Sciences.

## Author contributions

JC and WZ contributed to the conception of the study. YG, FW, and MY collected the samples. YG and HC performed the metagenomics analysis, analyzed the data, and wrote the manuscript. LL and PL revised the manuscript. All authors contributed to the article and approved the submitted version.

## Supplementary material

Supplementary Figure S1 The rarefaction curves of each sample in **(A)** 16S rDNA and **(B)** ITS sequencing.

Supplementary Figure S2 Relative abundances of the function of samples from green sea turtles and water samples. The function was predicted at level 1 of the KEGG pathway.

Supplementary Figure S3 Prediction of functional differences between samples from green sea turtles and water samples. The function was predicted at level 2 of the KEGG pathway. **(A)** Functional differences between carapacial samples from healthy and diseased green sea turtle. **(B)** Functional differences between gut microbes in diseased and healthy green sea turtles. **(C)** Functional differences between water samples from ponds with and without ulcerative carapacial disease.

Supplementary Figure S4 Bacterial species with significant abundance difference between each group. **(A)** Group of microorganisms in carapacial samples from healthy and diseased green sea turtle. **(B)** Group of the gut microbiome in diseased and healthy green sea turtles. **(C)** Group of microorganisms in water samples from ponds with and without ulcerative carapacial disease.

Supplementary Figure S5 Fungal species with significant abundance difference between each group. **(A)** Group of microorganisms in carapacial samples from healthy and diseased green sea turtle. **(B)** Group of microorganisms in water samples from ponds with and without ulcerative carapacial disease.

Supplementary Table S1 Sample information on green sea turtles and water.

Supplementary Table S2 Mean relative abundance of phylum, genus, and species in each group.

Supplementary Table S3 Permutational analysis of variance (PERMANOVA) between samples from healthy and diseased see turtles.
