# Supplementary figures and images for: Microbial composition of carapace, feces, and water column in captive juvenile green sea turtles with carapacial ulcers
Source: Front Vet Sci. 2022 Dec 15;9:1039519. doi: 10.3389/fvets.2022.1039519 (PMC9797667; doi:10.3389/fvets.2022.1039519)

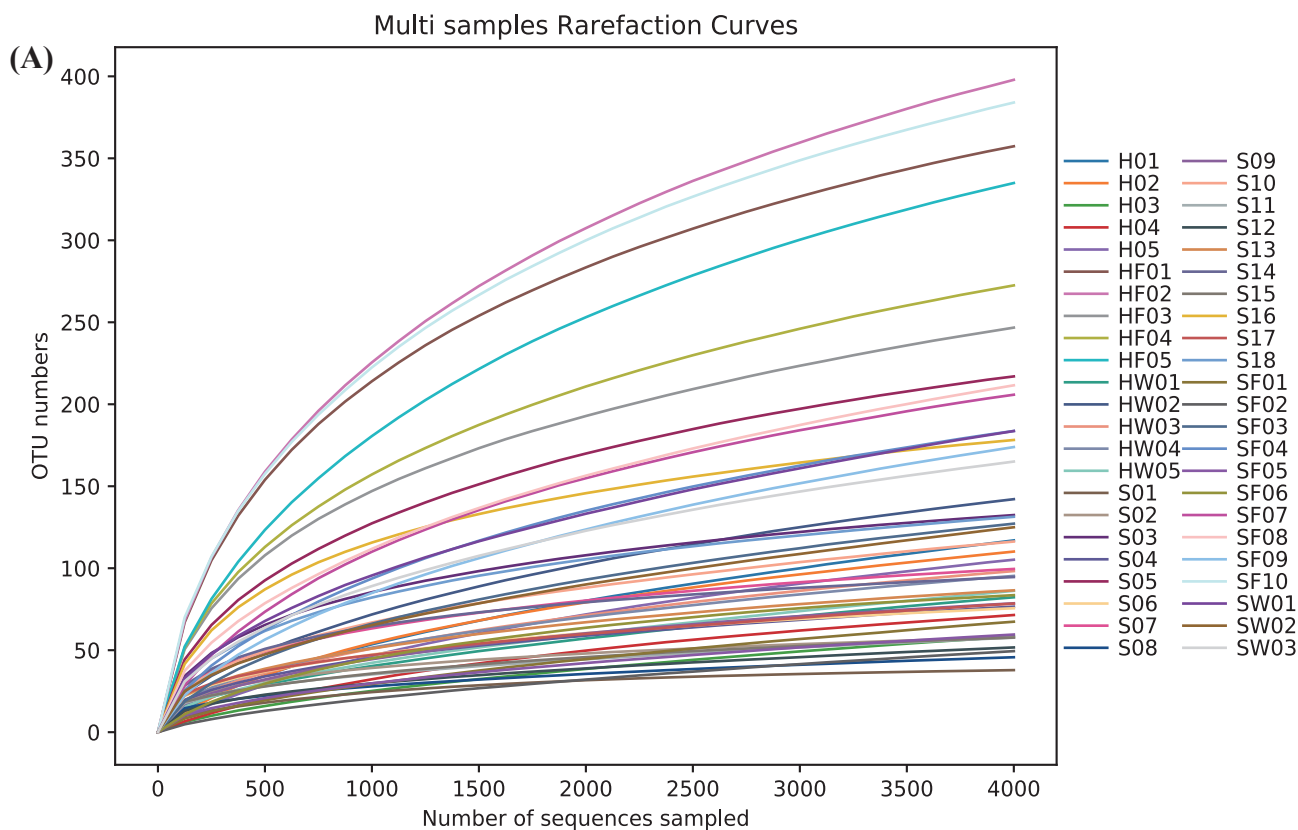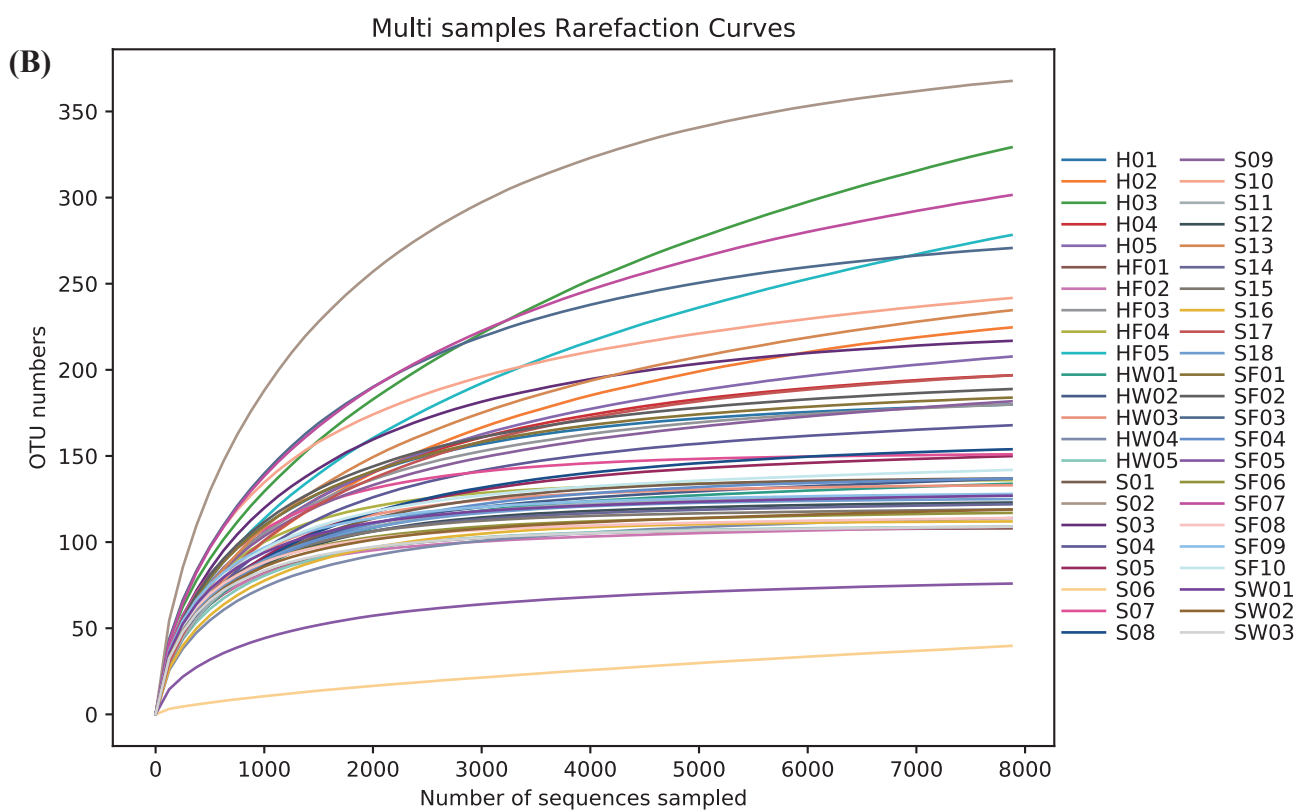

Supplement: Supplementary Figure S1 — The rarefaction curves of each sample in (A) 16S rDNA and (B) ITS sequencing. [file Data_Sheet_1.PDF]

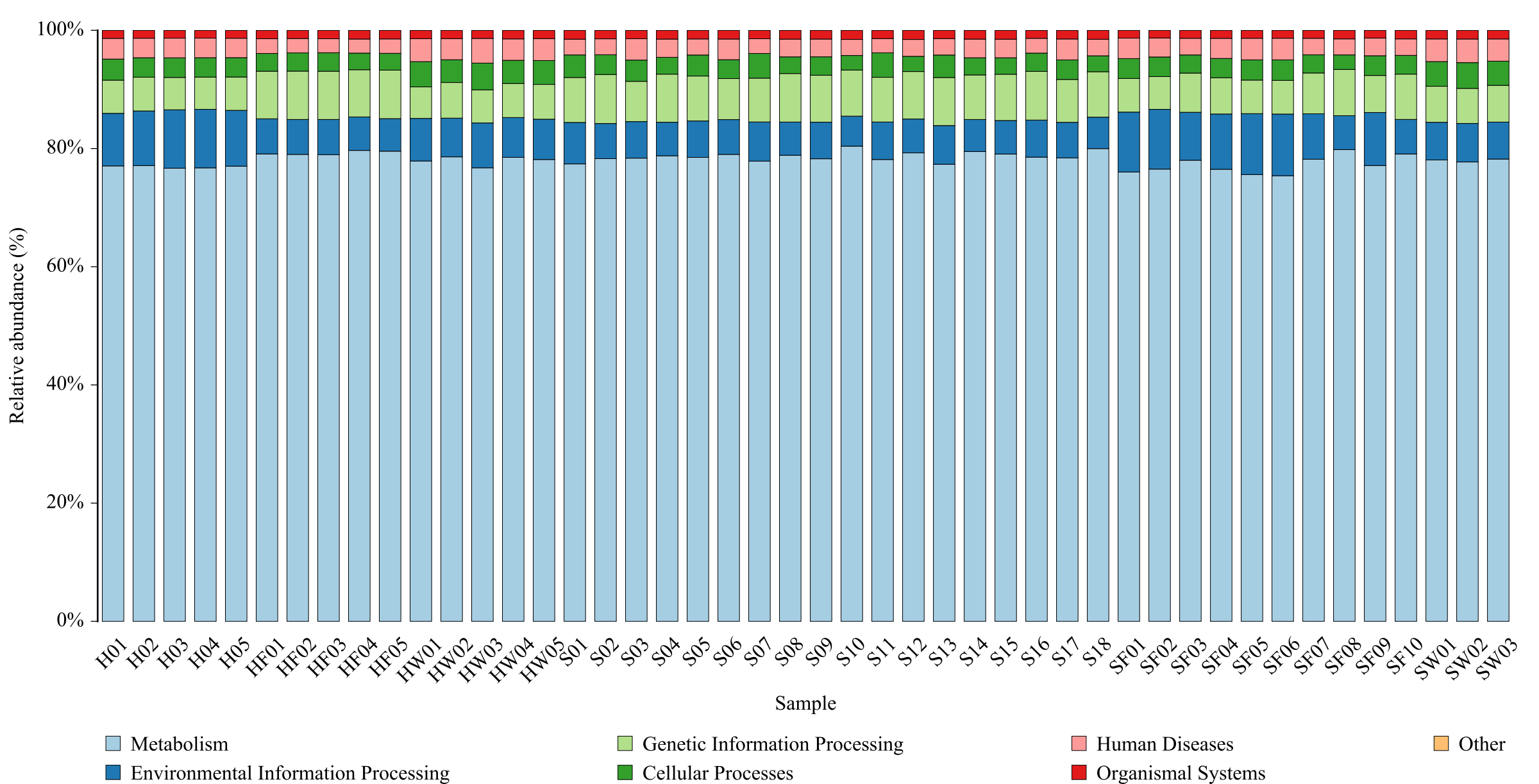

Supplement: Supplementary Figure S2 — Relative abundances of the function of samples from green sea turtles and water samples. The function was predicted at level 1 of the KEGG pathway. [file Data_Sheet_2.PDF]

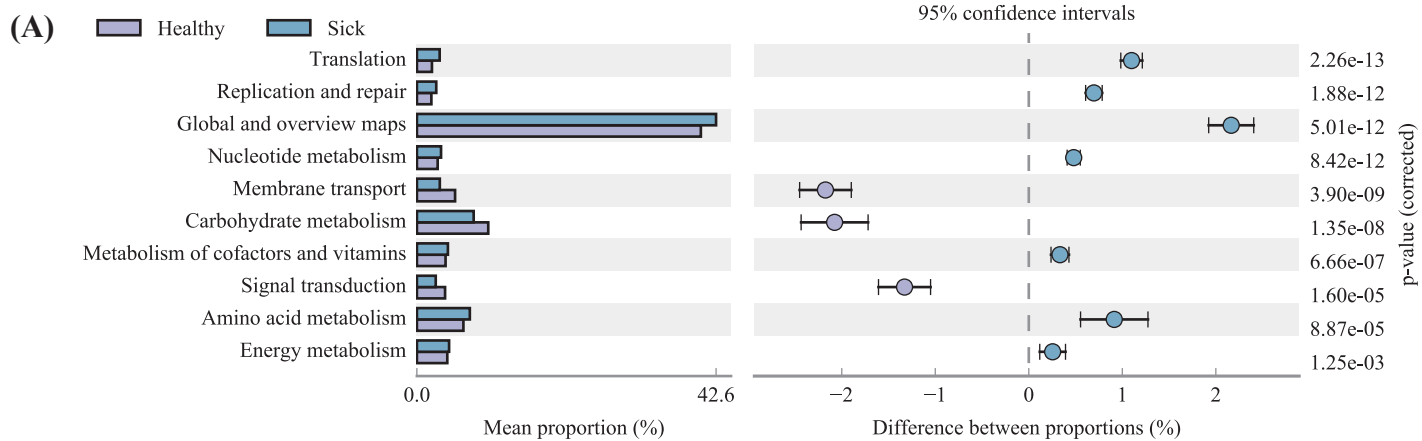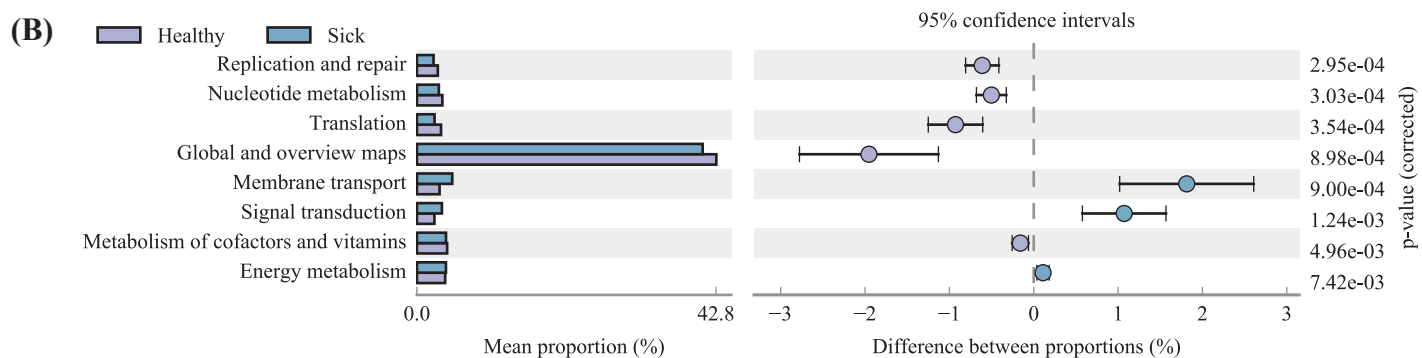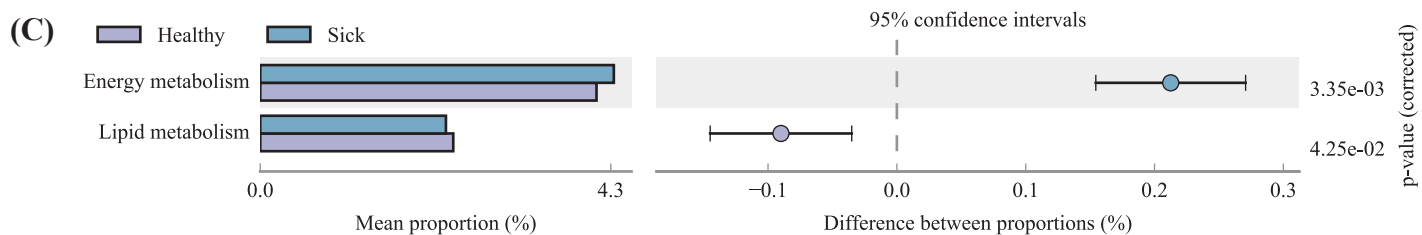

Supplement: Supplementary Figure S3 — Prediction of functional differences between samples from green sea turtles and water samples. The function was predicted at level 2 of the KEGG pathway. (A) Functional differences between carapacial samples from healthy and diseased green sea turtle. (B) Functional differences between gut microbes in diseased and healthy green sea turtles. (C) Functional differences between water samples from ponds with and without ulcerative carapacial disease. [file Data_Sheet_3.PDF]

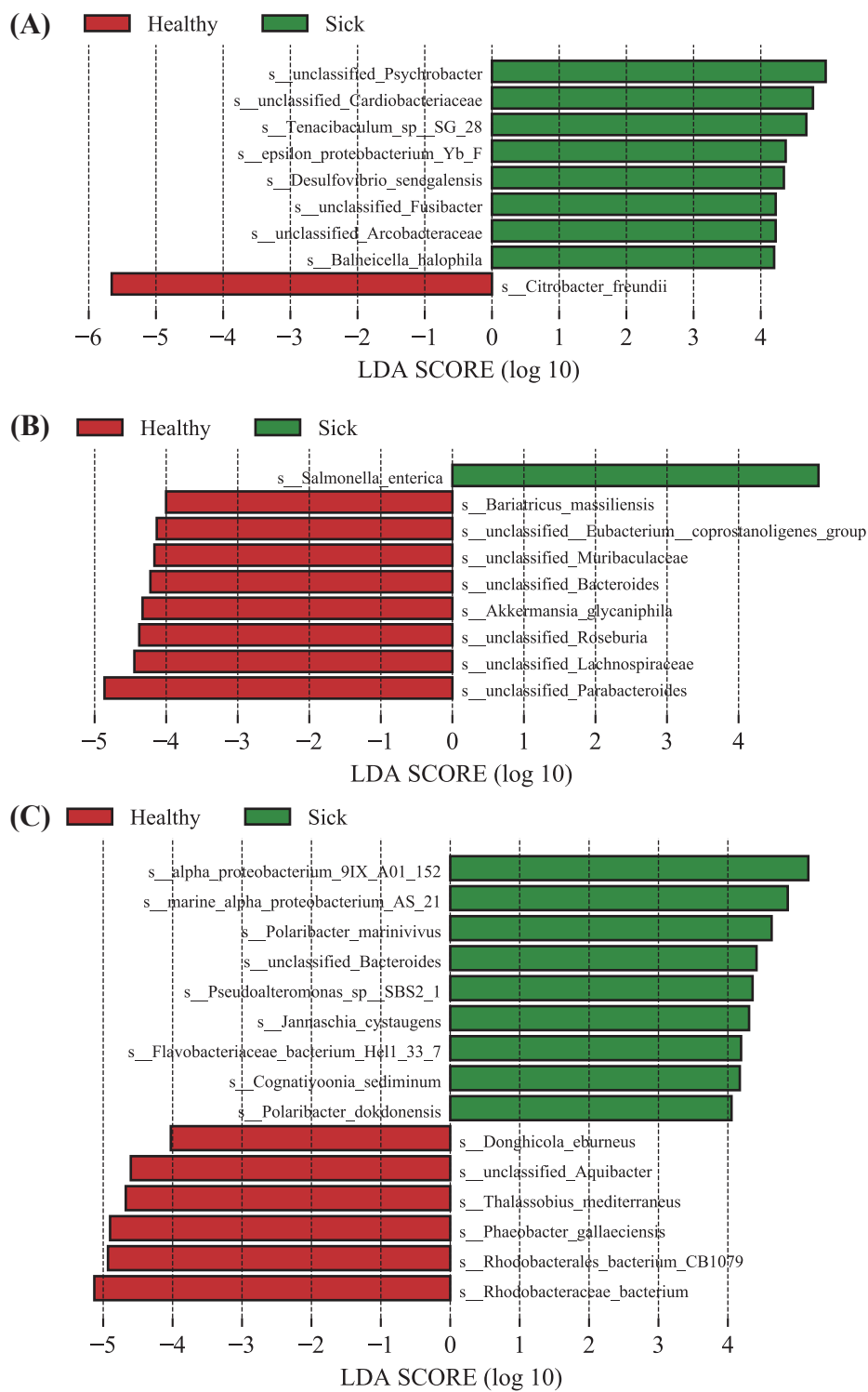

Supplement: Supplementary Figure S4 — Bacterial species with significant abundance difference between each group. (A) Group of microorganisms in carapacial samples from healthy and diseased green sea turtle. (B) Group of the gut microbiome in diseased and healthy green sea turtles. (C) Group of microorganisms in water samples from ponds with and without ulcerative carapacial disease. [file Data_Sheet_4.PDF]

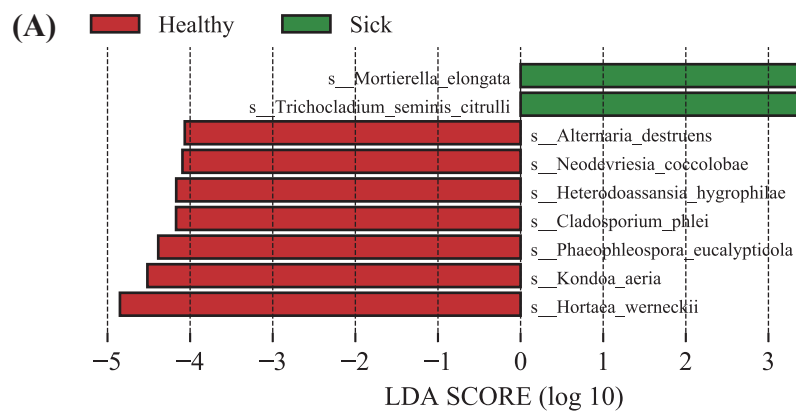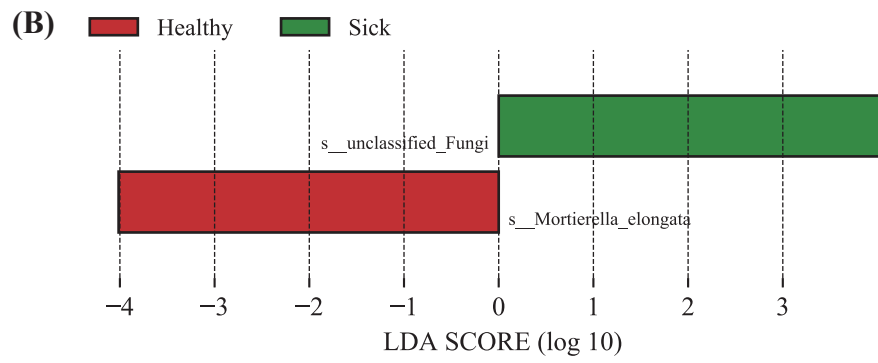

Supplement: Supplementary Figure S5 — Fungal species with significant abundance difference between each group. (A) Group of microorganisms in carapacial samples from healthy and diseased green sea turtle. (B) Group of microorganisms in water samples from ponds with and without ulcerative carapacial disease. [file Data_Sheet_5.PDF]
