# Supplementary material for: Microbial composition of carapace, feces, and water column in captive juvenile green sea turtles with carapacial ulcers
Source: Front Vet Sci. 2022 Dec 15;9:1039519. doi: 10.3389/fvets.2022.1039519 (PMC9797667; doi:10.3389/fvets.2022.1039519)
Supplement: Supplementary Table S1 — Sample information on green sea turtles and water. [file Table_1.DOCX]

STable S1 Sample information of high-throughput sequencing

| Group | Sample | Sample details | Sample date |
| --- | --- | --- | --- |
| Shell | H01 | Shell surface of a healthy juvenile green sea turtle | 2022.3.28 |
| Shell | H02 | Shell surface of a healthy juvenile green sea turtle | 2022.3.28 |
| Shell | H03 | Shell surface of a healthy juvenile green sea turtle | 2022.3.28 |
| Shell | H04 | Shell surface of a healthy juvenile green sea turtle | 2022.3.28 |
| Shell | H05 | Shell surface of a healthy juvenile green sea turtle | 2022.3.28 |
| Ulcer | S01 | Ulcerated tissue on the shell of a diseased juvenile green sea turtle | 2022.3.6 |
| Ulcer | S02 | Ulcerated tissue on the shell of a diseased juvenile green sea turtle | 2022.3.6 |
| Ulcer | S03 | Ulcerated tissue on the shell of a diseased juvenile green sea turtle | 2022.3.6 |
| Ulcer | S04 | Ulcerated tissue on the shell of a diseased juvenile green sea turtle | 2022.3.6 |
| Ulcer | S05 | Ulcerated tissue on the shell of a diseased juvenile green sea turtle | 2022.3.6 |
| Ulcer | S06 | Ulcerated tissue on the shell of a diseased juvenile green sea turtle | 2022.3.6 |
| Ulcer | S07 | Ulcerated tissue on the shell of a diseased juvenile green sea turtle | 2022.3.18 |
| Ulcer | S08 | Ulcerated tissue on the shell of a diseased juvenile green sea turtle | 2022.3.18 |
| Ulcer | S09 | Ulcerated tissue on the shell of a diseased juvenile green sea turtle | 2022.3.18 |
| Ulcer | S10 | Ulcerated tissue on the shell of a diseased juvenile green sea turtle | 2022.3.18 |
| Ulcer | S11 | Ulcerated tissue on the shell of a diseased juvenile green sea turtle | 2022.3.18 |
| Ulcer | S12 | Ulcerated tissue on the shell of a diseased juvenile green sea turtle | 2022.3.18 |
| Ulcer | S13 | Ulcerated tissue on the shell of a diseased juvenile green sea turtle | 2022.3.18 |
| Ulcer | S14 | Ulcerated tissue on the shell of a diseased juvenile green sea turtle | 2022.3.18 |
| Ulcer | S15 | Ulcerated tissue on the shell of a diseased juvenile green sea turtle | 2022.3.25 |
| Ulcer | S16 | Ulcerated tissue on the shell of a diseased juvenile green sea turtle | 2022.3.25 |
| Ulcer | S17 | Ulcerated tissue on the shell of a diseased juvenile green sea turtle | 2022.3.25 |
| Ulcer | S18 | Ulcerated tissue on the shell of a diseased juvenile green sea turtle | 2022.3.25 |
| Fecal | HF01 | Fecal of healthy juvenile green sea turtle | 2022.3.16 |
| Fecal | HF02 | Fecal of healthy juvenile green sea turtle | 2022.3.16 |
| Fecal | HF03 | Fecal of healthy juvenile green sea turtle | 2022.3.16 |
| Fecal | HF04 | Fecal of healthy juvenile green sea turtle | 2022.3.16 |
| Fecal | HF05 | Fecal of healthy juvenile green sea turtle | 2022.3.16 |
| Fecal | SF01 | Fecal of sick juvenile green sea turtle with ulcer | 2022.3.25 |
| Fecal | SF02 | Fecal of sick juvenile green sea turtle with ulcer | 2022.3.25 |
| Fecal | SF03 | Fecal of sick juvenile green sea turtle with ulcer | 2022.3.25 |
| Fecal | SF04 | Fecal of sick juvenile green sea turtle with ulcer | 2022.3.25 |
| Fecal | SF05 | Fecal of sick juvenile green sea turtle with ulcer | 2022.3.25 |
| Fecal | SF06 | Fecal of sick juvenile green sea turtle with ulcer | 2022.4.10 |
| Fecal | SF07 | Fecal of sick juvenile green sea turtle with ulcer | 2022.4.10 |
| Fecal | SF08 | Fecal of sick juvenile green sea turtle with ulcer | 2022.4.10 |
| Fecal | SF09 | Fecal of sick juvenile green sea turtle with ulcer | 2022.4.10 |
| Fecal | SF10 | Fecal of sick juvenile green sea turtle with ulcer | 2022.4.10 |
| Water | HW01 | Water of pool where ulcers were not found | 2022.3.8 |
| Water | HW02 | Water of pool where ulcers were not found | 2022.3.8 |
| Water | HW03 | Water of pool where ulcers were not found | 2022.3.8 |
| Water | HW04 | Water of pool where ulcers were not found | 2022.3.8 |
| Water | HW05 | Water of pool where ulcers were not found | 2022.3.8 |
| Water | SW01 | Water of pool where ulcers were found | 2022.3.9 |
| Water | SW02 | Water of pool where ulcers were found | 2022.3.9 |
| Water | SW03 | Water of pool where ulcers were found | 2022.3.9 |
