# Supplementary material for: Microbial composition of carapace, feces, and water column in captive juvenile green sea turtles with carapacial ulcers
Source: Front Vet Sci. 2022 Dec 15;9:1039519. doi: 10.3389/fvets.2022.1039519 (PMC9797667; doi:10.3389/fvets.2022.1039519)
Supplement: Supplementary Table S3 — Permutational analysis of variance (PERMANOVA) between samples from healthy and diseased see turtles. [file Table_3.DOC]

**Supplemental Table 3A** Permanova analysis of bacterial between healthy and sick samples

| Group | R^2^ | P-value |
| --- | --- | --- |
| Healthy water-Sick water | 0.540 | 0.019 |
| Healthy fecal-Sick fecal | 0.608 | 0.001 |
| Healthy shell-Shell ulcer | 0.481 | 0.001 |

**Supplemental Table 3B** Permanova analysis of fungus between healthy and sick samples

| Group | R^2^ | P-value |
| --- | --- | --- |
| Healthy water-Sick water | 0.265 | 0.019 |
| Healthy fecal-Sick fecal | 0.037 | 0.905 |
| Healthy shell-Shell ulcer | 0.353 | 0.001 |
